# The Prevalence of Gastroesophageal Reflux Disease in Azerbaijan: A Population-Based Cross-sectional Study

**DOI:** 10.5152/tjg.2023.211042

**Published:** 2023-11-01

**Authors:** Sevda Aghayeva, David Katzka, Nargiz Afandiyeva, Serhat Bor, Gulustan Babayeva, Alihuseyn Hidayatov, Gulay Mammadzada

**Affiliations:** 1 Division of Gastroenterology and Hepatology, Baku Medical Plaza Hospital, Baku, Azerbaijan; 2 Division of Gastroenterology and Hepatology, Mayo Clinic, Rochester, USA; 3 Division of Gastroenterology, National Oncology Center, Baku, Azerbaijan; 4 Division of Gastroenterology & Ege Reflux Study Group, Ege University, İzmir, Turkey; 5 Azerbaijan State Advanced Training Institute for Doctors named after A. Aliyev, Baku, Azerbaijan; 6 Division of Gastroenterology, Azerbaijan Medical University, Baku, Azerbaijan; 7 Division of Psychiatry, Azerbaijan Medical University, Baku, Azerbaijan

**Keywords:** Gastroesophageal reflux disease, GERD, prevalence, epidemiology, Azerbaijan

## Abstract

**Background/Aims:**

The prevalence of gastroesophageal reflux disease in Azerbaijan has not been evaluated before. The aim of our study was to determine the prevalence of gastroesophageal reflux disease based on the validated reflux questionnaire.

**Materials and Methods:**

A total of 1026 individuals from 7 regions of Azerbaijan were included in the cross-sectional study conducted via face-to-face administration of the validated Mayo Clinic’s gastroesophageal reflux disease questionnaire. Gastroesophageal reflux disease was diagnosed if an individual had heartburn and/or regurgitation occurring at least once a week.

**Results:**

The prevalence of gastroesophageal reflux disease in Azerbaijan was 22.7% with significant female predominance (26.1% vs. 15.3%; *P* < .0001). Gastroesophageal reflux disease was prevalent in 17% of those aged below 35 years; 22.7% of those in the age range 36-55 years, and 38.5% of those who are above 56 years, which, accordingly, indicates that gastroesophageal reflux disease becomes significant as age increased (*P* < .0001). Male respondents younger than 35 years had much lower rates of gastroesophageal reflux disease than in older groups (5.7% vs. 22.7%, *P* < .0001), whereas in females older than 55 years, age was a significant factor for increasing gastroesophageal reflux disease symptoms (22.6% vs. 50%, *P* < .001). Reflux was observed in 18.1% of normal-weight respondents (body mass index 18.6-24.9), 25.6% of overweight (body mass index 25-29.9), and 30.4% of obese (body mass index > 30) individuals (*P* = .001). Regarding marital status, the prevalence was the lowest in the single subjects’ group (17%), close to average in the married group (23.8%), and the highest (41.7%) in divorced/widowed cases (*P* = .003). Stress significantly affected the gastroesophageal reflux disease distribution, affecting 59.4% of all respondents (*P* < .004).

**Conclusion:**

Gender, body mass index, increased age, marital status, and stress were precipitating factors of gastroesophageal reflux disease. Socioeconomic diversity, along with lifestyle/habits, did not play a crucial role in the gastroesophageal reflux disease prevalence distribution.

**Main Points**

- This is the first cross-sectional study of gastroesophageal reflux disease (GERD) prevalence in the Transcaucasian region; thus, neighboring countries may use is it as a keystone for further research projects.
- The prevalence of GERD in Azerbaijan was significantly higher in women compared to men, yet men had more severe symptomatic manifestations.
- Body mass index, age, marital status, and stress played a crucial role in the GERD prevalence distribution.

## Introduction

Gastroesophageal reflux disease (GERD) is one of the most common chronic diseases in adults which affects 5% of the Asian and 10%-20% of the Western population.^1^ It is considered a multifactorial process, as various environmental, dietary, genetic, and stress-related factors may contribute to its pathophysiology.^2^ As described in epidemiological studies, GERD can be diagnosed based on common symptoms of heartburn and acid regurgitation.^3^ With the broad use of prescribed and over-the-counter proton pump inhibitors (PPIs), antacids, and histamine-receptor antagonists (H2RA), complications such as bleeding, erosive esophagitis, or peptic stricture are becoming less common.^4^ However, continuous medication use, dietary restrictions, and recommended lifestyle modifications impact the quality of life in affected individuals.^5^ As a result, the estimation of the prevalence of GERD in varied geographic populations is important. This is particularly cogent as most studies of GERD appear to derive from Western European, American, and East Asian populations. To assess the prevalence in large population studies without an invasive diagnostic method, many investigators have been using various questionnaires.^6-8^

Azerbaijan is located at the junction of Europe, Asia, and Middle East, occupying the Greater Caucasus mountains in the northern part of the country and the Lesser Caucasus in the southern part. With a population of over 10 million, it is considered the most populous country of the region, with a vast majority of the represented race being Caucasian. The Caucasus sub-region is comprised of Azerbaijan, Armenia, Georgia, as well as a part of Russia. Throughout its history, Caucasus was conquered by various empires, including the Achaemenid, Neo-Assyrian Empire, Parthian, Roman, Sassanian, Byzantine, Mongol, Ottoman, Iranian, and Russian, thus representing a fusion of their faiths and extreme cultural and linguistic differentiation, with more than 50 diverse autochthonous ethnic groups.^9^

Over the past century, Azerbaijan was a part of Soviet Union, which historically grouped 15 countries, based on administrative classification and geographic location, shared common history leading to anthropological similarities in their societies, economic growth, and distinctive genetic features. Since achieving independence in early 1990s, Azerbaijan has undergone a rapid transition, including significant emigration from rural areas and dietary changes. Taking into consideration the high-risk factors for GERD, including national cuisine rich with fatty meals and frequent consumption of hot tea, as well as the increasing prevalence of comorbidities, such as obesity, alcoholism, and stress-related disorders, the evaluation of GERD is crucial for timely prevention and treatment. This evaluation could also yield new insights into GERD as a result of the melting pot of the surrounding national influences. Population-based data on the prevalence and distribution of GERD in former Soviet countries are very scarce. The epidemiology of GERD in Azerbaijan also remains unstudied. Contrasting the prevalence of and risk factors for GERD in this region with that in Western Europe and the USA may help to understand the contribution of genetic versus environmental factors to GERD in the Caucasian population.

Our study will be the first published population-based prevalence research among post-Soviet countries in the Transcaucasian region. It might also be a paradigm for the further evaluation of GERD in the bordering countries.

## Materials and Methods

### Study Design

This questionnaire-based cross-sectional study was performed in Azerbaijan between January and December 2018. Based on the data on total population of 9 949 534, the sample size was estimated based on a power of 80%, assuming a GERD prevalence of 20%, with 1% standard error (19%-21%) of CI and exclusion rate of 7.5% and defined as 1000 subjects. The level of significance was defined as *P* < .05.

A population-based study was conducted in 7 different regions of Azerbaijan involving 1026 adults older than 18 years of age. This sample size represented the national population in terms of age, gender, and geographic location. For each geographic region, 1 major city was selected. The cities were selected taking into account the representative characteristics of the area with a population of over 50 000. Exclusion criteria were previous gastric or esophageal surgery, any type of current cancer except non-melanoma skin cancers, pregnancy, psychiatric disease, dementia, and refusal to participate in the study.

Trained interviewers, such as physicians and medical residents, randomly conducted face-to-face interviews of the passers-by near the so-called “ASAN service”—a service that brings together representatives of 10 governmental entities and private companies providing services in a public–private partnership with more than 300 services provided, including birth, death, and marriage registration; receiving and changing identity cards, passports, and driver licenses; managing real estate records, immigrant status, and other civic services; as well as banking, insurance, legal support, and translation. These services are located in all regions of Azerbaijan and are equally visited by all the segments of population in terms of gender, age, social status, and income. Hence, the interviewed residents represent both rural and urban population. The number of respondents in each region was calculated according to population of region over population of the country.

### Questionnaire

The original GERD questionnaire developed by Locke et al^10^ was used in our study. It was received from Mayo Clinic with reserved copyright. The questionnaire contained 80 questions. The approximate time in which the survey was completed was 20-25 minutes. The questionnaire included major symptoms, such as heartburn and regurgitation, as well as related symptoms, such as dyspepsia, dysphagia, odynophagia, and chest pain. Past medical history, family history, demographic and socioeconomic data, age, weight, height (shown in the identification card), employment, level of education, marital status, and recent stress level were assessed during the interview. Past medical history assessment including upper and lower gastrointestinal symptoms; ear-nose-throat specialist (ENT), respiratory, and cardiac problems; the number of physician visits and performed diagnostic procedures related to the upper gastrointestinal symptoms, medication usage; previous pregnancies; smoking; and alcohol/tea/coffee consumption was recorded.

Height and weight were measured according to a standard protocol. Body mass index (BMI) was calculated as body weight in kilograms divided by the square of height in meters (kg/m^2^) and classified using the WHO classification as underweight (lower than 18.5), normal (18.5-24.9), overweight (25-29.9), and obese (30 or more).

Positive cigarette consumption was defined as at least once per week for the past 6 months, and positive alcohol use was defined as drinking once per month for the past 6 months.

The questionnaire was linguistically validated by independent translation from English to Azeri by 3 local interpreters and back-translated by 3 native-English speakers, fluent in Azeri language. The authors discussed, modified any discrepancies in translation, and adapted the questionnaire to cultural terminology. Taking into consideration that there is no exact translation for the word “reflux,” a definition of the symptom was added to the questions. Patients are asked about the frequency/severity of “burning behind breastbone” and “pain behind breastbone” for heartburn as well as “movement of acid or gastric contents to the throat” and “acid taste in the mouth” for regurgitation. The final version was reviewed by the authors.

Feasibility of the questionnaire was assessed based on the percentage of missing values and unanswered questions, difficulty ratings, and time spent. Test–retest reliability was analyzed for each respondent using the Cohen’s kappa coefficients in 50 off-PPI individuals who repeated the interview 4 weeks after taking the first survey. The correlation between test and retest was analyzed via Pearson’s coefficient.

The reliability of the Azerbaijani version was descent with Cronbach’s alpha values higher than 75% for major symptoms in the period of 12 months of prevalence. The accuracy of the questionnaire data was rechecked by a telephone call of a random 20% of the respondents.

The reliability and validity analysis was performed by interviewing 50 patients who were endoscopically diagnosed with GERD and a control group with no reflux symptoms. Gastroesophageal reflux disease was defined if the subject had a heartburn and/or regurgitation once a week or more often. An episode of 1 of the major symptoms occurring at least once a week was defined as a “frequent symptom.” A major symptom occurring less than once a week within the last 12 months was defined as an “occasional symptom.” Following detailed explanation of the study purpose, written informed consent was obtained from every individual in accordance with the ethical principles stated in the Declaration of Helsinki and this study was approved by the institutional ethics committee of Azerbaijan State Advanced Training Institute for Doctors named after A. Aliyev (Approval No: 002, date: January 12, 2019).

### Statistical Analysis

We have used the Statistical Package for the Social Sciences (IBM Corp.; Armonk, NY, USA) version 25.0 to analyze the data. Study groups were analyzed by comparing the “GERD-present” and “GERD-absent” groups, followed by comparing “never,” “occasional,” and “frequent” groups. Categorical variables were shown as frequency tables. The Student’s *t*-test was used for the comparison of numerical data of independent groups, along with Kruskal–Wallis nonparametric analysis of variance used for global comparisons of 3 groups. Cross-table statistics was used for group comparison; significance level was analyzed by the chi-squared test. Data are expressed as mean (SD), prevalence as percent, and median with 95% CI as appropriate. The statistical significance level was defined at *P* < .05.

## Results

### Demographics

A total of 1267 subjects were interviewed during the evaluation period. After excluding 241 subjects with incomplete questionnaires, 1026 individuals [654 (63.7%) females, mean age ± SD 39.7 ± 13.3] were included in the study. Less men were available for interviews due to inability to arrange a research time or leaving Azerbaijan for employment.

The general characteristics of the respondents are summarized in Table 1.

Based on the questionnaire answers, GERD was diagnosed in 22.7% of overall population. The overall prevalence of GERD was 26.5% for females and 15.7% for males (*P* < .0001).

**Figure 1.**
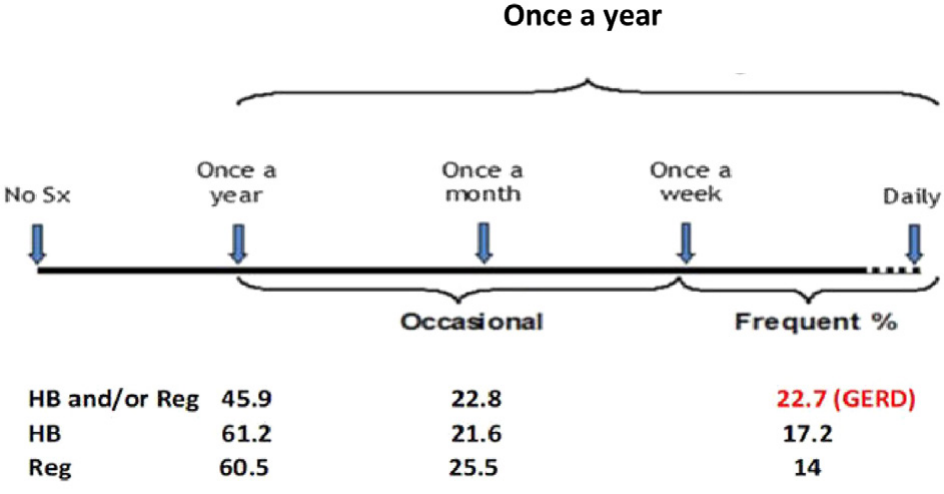
The prevalence of major gastroesophageal reflux disease (GERD) symptoms: heartburn (HB) and regurgitation (Reg).

Frequent and occasional symptoms were noted in 17.2% vs. 21.6% of interviewed male population within the last 12 months for the heartburn-only group in contrast to 14% vs. 25.5% for the regurgitation group, respectively. Among female respondents, frequent and occasional heartburn was noted in 27.75 vs. 13.6%, while the regurgitation percentage was 8.4% vs. 28.4%. The frequency of major and additional symptoms is shown in Figure 1 and Table 2.

In a group of respondents who gave positive answers for both heartburn and regurgitation questions, age was a statistically notable factor (20.1% in 35 and below age group; 21.6% in the 36-55 age group, and 27% in the 56 and above group; *P* = .003).

**Figure 2.**
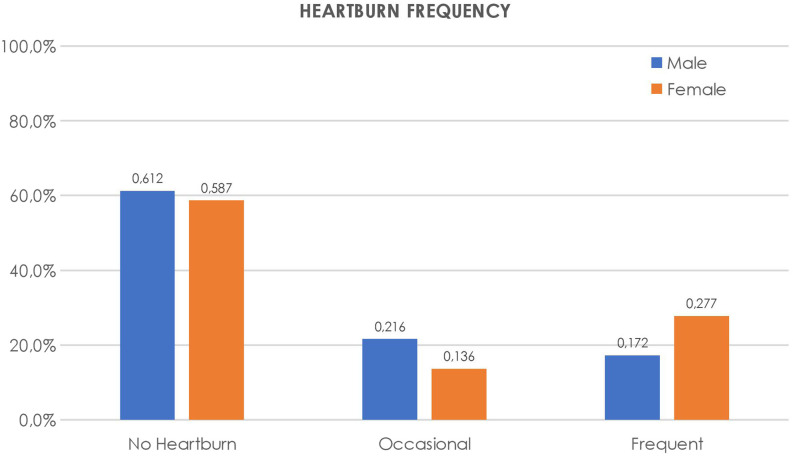
Frequency of heartburn in males and females.

**Figure 3.**
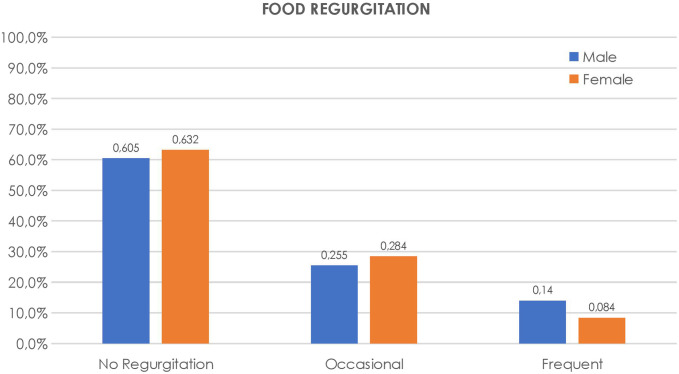
Frequency of food regurgitation rates in male and female groups.

The frequency of heartburn and regurgitation was compared by gender and is shown in Figures 2 and 3.

**Figure 4.**
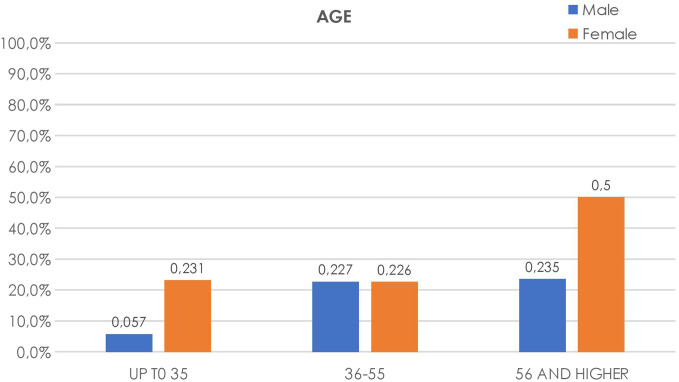
Gastroesophageal reflux disease prevalence distribution in age groups, varying by gender.

Age-based evaluation of questionnaire answers revealed that there was a significant association between GERD and various age groups, indicating higher prevalence in older age groups even in weighted age-adjusted data. The mean ± SD age of cases with occasional heartburn was 37 ± 11.94 years, whereas the mean ± SD age of cases with frequent symptoms was 45.08 ± 13.15 years (*P* < .0001). For regurgitation, the difference between occasional and frequent symptoms was not statistically significant (*P* = .381). Regarding patients who suffered from both heartburn and regurgitation, age was not an impacting factor (*P* = .02); however, the occurrence of GERD in women was twice more than that of men (*P* = .003), as shown in Figure 4.

**Figure 5.**
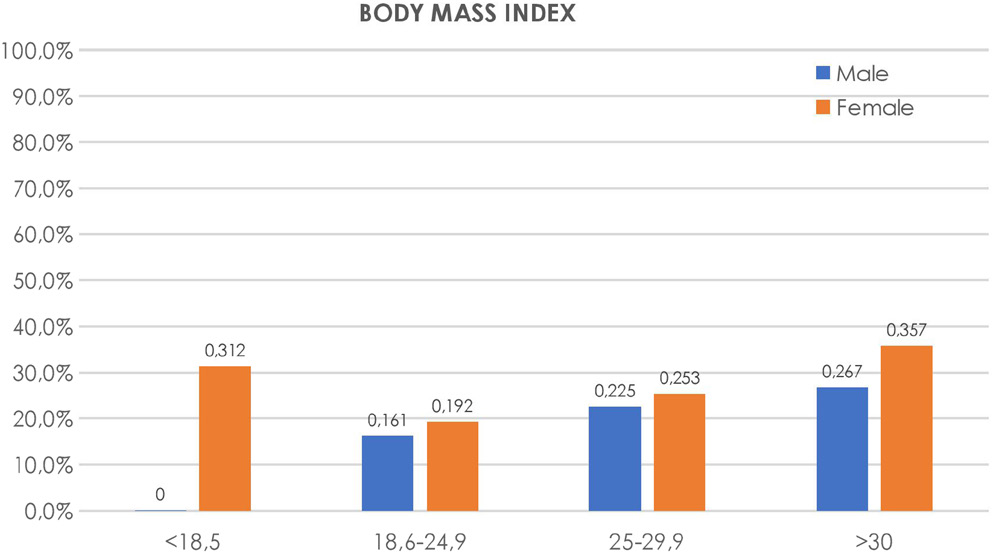
Gastroesophageal reflux disease prevalence distribution in different weight groups, varying by gender.

Gastroesophageal reflux disease was prevalent in 17% of those aged below 35 years; 22.7% of those in the age range 36-55, and 38.5% of those who are above 56 years. Gastroesophageal reflux disease prevalence was significantly greater with increasing age (*P* < .0001). As seen in Figure 5, the lowest prevalence of GERD was shown in male respondents younger than 35 years (5.7% vs. 22.7%, *P* < .0001), whereas increasing symptoms were observed in female patients aged older than 55 years (22.6% vs. 50%, *P* < .001).

Additionally, another factor that influenced GERD prevalence was BMI. Reflux was observed in 18.1% of normal-weight respondents (BMI 18.6-24.9), 25.6% of overweight (BMI 25-29.9), and 30.4% of obese (BMI > 30) individuals (*P* = .001). Interestingly, in the underweight (BMI <18.5) group, all 35 respondents were females, 31.2% of whom were diagnosed with GERD (*P* < .001). More than one-third (36.2%) of the interviewed individuals with GERD reported similar symptoms in their first-degree relatives (*P* > .0001).

**Figure 6.**
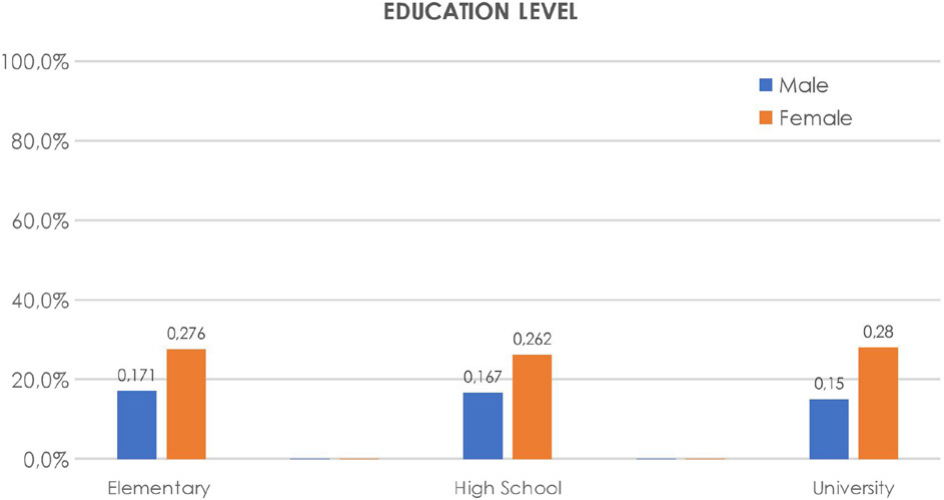
Gastroesophageal reflux disease prevalence distribution according to educational level, varying by gender.

The level of education and status of employment did not affect the prevalence of the disease. Both males and females showed minor differences in terms of education and academic degree (*P* = .38); however, female predominance was noticed in all groups (*P* < .005). Gastroesophageal reflux disease prevalence regarding education level is shown in Figure 6.

**Figure 7.**
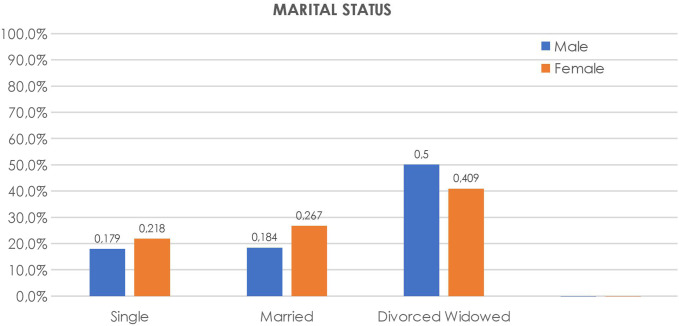
Gastroesophageal reflux disease prevalence distribution according to marital status, varying by gender.

Data concerning the marital status impacted the frequency of symptoms among groups, which is shown in Figure 7. The prevalence was the lowest in the single subjects’ group (17%), close to average in the married group (23.8%), and the highest (41.7%) in divorced/widowed cases (*P* = .003).

As many as 40.4% of male respondents consumed alcohol and 53.1% smoked, while only 6.2% of women consumed alcohol and 2.4% used tobacco products (*P* < .0001).

Despite obvious male-to-female ratio disparity, among all patients with GERD, 19.7% consumed alcohol compared to 18.3% of subjects with no GERD; thus, alcohol use was not considered a statistically significant association regardless of age and gender (*P* = .85).

Even though the Azerbaijani population consumes mostly hot tea, coffee drinkers did not show more prevalent GERD symptoms (*P* = .14).

Stress significantly affected the GERD distribution, affecting 59.4% of all respondents (*P* < .004).

Since there is a lack of governmental insurance program in Azerbaijan, patients often skip doctor visits and relieve GERD complaints with over-the-counter acid-lowering medications which are relevantly affordable. Thus, individuals with GERD had a higher tendency of consuming acid-lowering medications (29.9% vs. 3.6%; *P* < .0001).

Furthermore, positive reflux symptoms were reported in 25% of respondents taking non-steroidal anti-inflammatory drugs vs. 13.9% of those who do not (*P* = .05). Medication use and lifestyle habits along with their association with GERD are shown in Tables 3 and 4.

## Discussion

Gastroesophageal reflux disease is a global disease that is increasing in prevalence. It is manifested in various symptoms, resulting in the impairment of the individual’s quality of life, injury, or complications. This is the first population study to evaluate the prevalence of GERD in Azerbaijan. We demonstrated that changes in dietary pattern, increasing socioeconomic stress level, the presence of concomitant medical conditions, as well as certain lifestyle and habits have been postulated to affect the prevalence of GERD in developing countries.

Our findings were similar to the rates of GERD studied in the neighboring regions, such as Turkey (22.8%), Iran (28%), and Moscow (23.6%), where a similar questionnaire, criteria, and methodology were implemented.^11-13^ Data from other former Soviet countries bordering Azerbaijan are lacking. A recent meta-analysis by Nirwan et al ^14^ conducted in 2020 reveals substantial variations in the GERD prevalence globally, with the lowest rate being reported in China (2.5%)^15^ and the highest in a study conducted in Saudi Arabia (45.4%).^16^ This review also identified the countries with the highest and lowest pooled prevalence of GERD as Turkey [22.40% (95% CI 18.53%-126.5%)] and China [4.16% (95% CI 3.35%-15.05%)], respectively. This fact highlights the disparity in the prevalence of GERD between sub-regions within the same continent. It was especially evident in Asia where the East Asia sub-region showed the lowest GERD prevalence and the Middle East showed the highest prevalence. Yet, it is still poorly understood why some individuals have more frequent or severe symptoms as well as complications of reflux than others.

Based on the previously conducted research, Table 5 shows the pooled prevalence of GERD distribution in Azerbaijan compared with its bordering countries, as well as the USA and some of the European and Asian countries with computable number of studies.

One of the important questions in GERD is how much of the etiology is dependent on genetic vs. lifestyle factors. As Azerbaijan is almost exclusively a Caucasian population yet a geographically and culturally distinct area compared to other Western populations, this analysis may be important for assessing the role of race and GERD. Our data are similar to Western Europe and the USA. A systematic review by El-Serag et al^17^ found that the range of GERD prevalence estimates was 18.1%-27.8% in North America, 8.8%-25.9% in Europe, 2.5%-7.8% in East Asia, 8.7%-33.1% in the Middle East, 11.6% in Australia, and 23.0% in South America. In the USA, the prevalence was noted to range from 18.1% to 27.8%, similar to our findings in Azerbaijan. This is in contrast to Eastern populations such as China and Japan. Interestingly, the occurrence of GERD in the Azerbaijan population was independent of key lifestyle factors such as smoking and the use of alcohol which have been shown to be important contributors to GERD in the USA. It was also shown that the prevalence of GERD did not increase with increasing age above the age of 36 years in men. This is in contrast to GERD in men in Western populations. As a result, our data emphasize the important common denominator of race in populations where GERD is highly prevalent regardless of some of the lifestyle and demographic factors associated with GERD.

Our study also demonstrated clear differences between the manner in which GERD affects men and women. According to our outcomes, female predominance was significantly higher among GERD cases. Several epidemiologic studies have analogically reported that females have a tendency of having GERD more frequently than males.^11-13,18,19^ Our results are also supported by Eusebi et al^20^ who estimated the pooled prevalence of GERD symptoms to be marginally higher in women compared with men [16.7% (95% CI 14.9% to 18.6%) vs. 15.4% (95% CI 13.5% to 17.4%)], especially in the Middle East women are approximately 40% more likely to report GERD symptoms than men.

Our data also support the concept of different phenotypes of GERD in men and women.^21^ For example, along with respondents with BMI above 30, underweight (BMI <18.5) women but not men frequently had GERD symptoms (*P* > .001). Specifically, we have found that all underweight respondents were females, 83.3% of whom were below the age of 35 years. Previously, several studies have also reported the association of low BMI with more severe GERD symptoms in women. Kim et al^22^ reported that having a low BMI (<18.5 kg/m^2^) was associated with a high prevalence of GERD among Korean young women. In addition to that, Hongo et al^23^ showed that quality of life (QOL) in underweight GERD patients tended to be more severe than in normal-weight patients. This is in contrast to men where a majority of previous studies reported that high BMI was linked to a higher number of GERD symptoms.^24-28^ Further differences between men and women are suggested by the difference in symptoms. Whereas women were more likely to have symptomatic heartburn, men were likely to have regurgitation.^29^ These symptoms explain different mechanisms—whereas women express symptoms reflecting a greater sensitivity to esophageal acid exposure, men have more volumetric reflux.^30^ Mechanistically, women are more susceptible to GERD complaints, possibly resulting from a greater esophageal sensitivity in women than men.^22^ Men, on the other hand, more likely develop reflux from associated obesity linked to overeating, increased abdominal pressure,^31^ increased gastric distension,^32^ and eliciting transient lower esophageal sphincter (LES) relaxations and/or leading to LES incompetence^33^ and hiatal hernia;^34^ additionally, metabolic sequelae of central obesity may contribute to increased permeability of distal esophageal epithelium indicative of a perturbed epithelial barrier.^24^ Our study also found that only 6.2% of women consumed alcohol compared to the 40.4% rate in male respondents. Similarly, 6.2% of females and 53.1% of males smoked. Although our data did not show an overall difference in these factors compared to patients without GERD, it supports the likely greater additive effects of more severe GERD in Azerbaijan men.

Our study also explored the role of stress and marital status on GERD symptom prevalence. Stress was significantly associated with a higher prevalence of GERD (*P* > .001). It has been shown that subjects who have been exposed to life stressors are more likely to complain of symptoms of GERD^35^ and have greater sensitivity to esophageal acid exposure,^36^ while, on the other hand, the presence of troublesome GERD symptoms may affect one’s daily quality of life and can thereby induce stress.^37^ In the analysis of marital status, the prevalence of GERD was the lowest in the single subjects’ group (17%) and the highest (41.7%) in divorced/widowed cases (*P* = .003). Our results are similar to a Russian study which showed the lowest rate of 20.6% in the married group and the highest rate of 31.6% among divorced/widowed cases.^13^ A global systematic review with a meta-analysis of GERD prevalence and risk factors, stratified according to marriage status, also demonstrated that the highest prevalence was noted in divorced/separated/widowed individuals [22.95% (95% CI 13.19%-134.38%)] followed by married individuals [15.98% (95% CI 10.48%-122.35%)], and the lowest rate was seen in single individuals [12.85% (95% CI 6.59%-120.71%)].^14^ Whether the effect of marital status on the presence of GERD symptoms is due to associated lifestyle factors is unclear. Our study revealed a higher prevalence of GERD rate in first-line relatives, which may contribute to genetic predisposition of the disease. However, this data might be biased due to the selectivity in perception. In other words, if one of the relatives has GERD, and others hear complaints and know about the disease, they can recognize it more easily. Mohammed et al^38^ performed a twin study in a UK cohort, showing that 43% (95% CI 32%-55%) of the variance in liability to GERD is due to additive genetic factors.^38^ Cameron et al^39^ had obtained a similar result in a Swedish twin cohort, indicating that genetic rather than shared environmental effects contribute to GERD.

There were several limitations to this study. The most important limitation was that this study was based on data obtainable from a questionnaire without objective evidence of GERD such as endoscopy. As a result, the association of factors with Barrett’s esophagus or erosive esophagitis could not be determined. On the other hand, regurgitation as a symptom of reflux is less responsive to medical treatment and more likely associated with greater LES incompetence. Thus, we could postulate that men expressed a more severe phenotype of GERD compared to women. Female predominance among respondents was also a factor, limiting the quality of the article, since for various reasons, the majority of interviewed were females. There could also be bias in subject participation in this study as those with symptoms might have been more willing to participate. On the other hand, there were several strengths. Our study included a large and broad geographic population of Azerbaijan, supporting that our data represent the country’s population. We also used a validated reflux questionnaire with a systematic collection of demographic factors and BMI. The validity of our data is corroborated by similar results found in studies from other Western nations.

Azerbaijan is an interesting region, located at the junction of Europe and Asia, fusing Muslim culture with 70-year-long Soviet influence. The prevalence of GERD in Azerbaijan was significantly higher in women compared to men, yet men had more severe symptomatic manifestations of GERD. Body mass index, age, marital status, and stress played a crucial role in the GERD prevalence distribution. More accurate, comprehensive, and detailed endoscopic and motility examinations are needed in order to estimate the burden of GERD, morbidity prevalence, and its complication rates in the Azerbaijani population.

## Figures and Tables

**Table 1.** Demographic Characteristics of the Respondents

Characteristics	n (%)
Gender Male Female	372 (36.3) 654 (63.7)
	Age (years) <35 36-55 ≥56	482 (47) 337 (32.8) 207 (20.2)
Marital status Married Single Divorced/widow		666 (64.9) 288 (28.1) 72 (7.0)
		Education level University Specialized school High school Primary school Uneducated	521 (50.7) 176 (17.3) 307 (29.9) 15 (1.5) 7 (0.6)
	Body mass index (BMI) Underweight (<18.5 kg/m^2^) Normal (18.6-24.9 kg/m^2^) Overweight (25-29.9 kg/m^2^) Obese (>30 kg/m^2^)		36 (3.5) 594 (57.9) 258 (25.1) 138 (13.5)
Lifestyle factors/habits Smoking Coffee Alcohol Stress			204 (19.9) 534 (52.0) 324 (31.6) 238 (23.2)
			Percent of general population by the region Absheron Ganja-Qazakh Shaki-Zaqatala Lankaran Guba-Khachmaz Aran Shirvan	340 (33.1) 167 (16.3) 92 (9) 105 (10.2) 91 (8.9) 142 (13.8) 89 (8.7)

**Table 2.** Prevalence and Frequency of Additional Symptoms

	Regurgitation/Heartburn			
	None n (%)	Occasional n (%)	Frequent n (%)	*P*
Dysphagia	15.0%^‡§^	30.6%^†§^	32.3%^†‡^	<.001
Odynophagia	0.7%^‡§^	8.4%^†§^	19.4%^†‡^	<.001
Globus	1.6%^‡§^	17.6%^†§^	22.1%^†‡^	<.001
Cough	3.6%^§^	18.2%^†§^	19.6%^†‡^	<.0009
Chronic pharyngitis	25.8%^‡§^	37.6%^†§^	53.2%^†‡^	<.001
Hoarseness	1.1%^§^	4.9%^§^	6.4%^†^	<.001
NCCP	16.2%^‡§^	35.1%^†^	38.8%^†^	<.001
Belching/Burping	14.2%^‡§^	33.2%^†§^	36.5%^†‡^	<.001
Nausea	6.1%^‡§^	15.5%^§^	34.8%^†‡^	<.001
Vomiting	1.8%^‡§^	6.3%^§^	19.7%^†‡^	<.001
Hematemesis	0.1%^‡§^	0.9%^†§^	1.5%^†‡^	<.001
Hiccups	0.9%^§^	4.4%^§^	5.9%^†^	<.001

^†^Different from the “none” group.

^‡^Different from the “rare” group.

^§^Different from the “frequent” group.

NCCP, noncardiac chest pain.

**Table 3.** Lifestyle Factors/Habits and Their Association with GERD

	GERD	No GERD	*P*
Smoking	165 (22.1%)	30 (14.3%)	.18
Coffee	135 (61.6%)	390 (51.4%)	.14
Alcohol	36 (19.7%)	123 (18.3%)	.85

GERD, gastroesophageal reflux disease.

**Table 4.** Medication Use and Its Association with GERD

	GERD	No GERD	*P*
PPI/antacids	60 (29.9%)	21 (3.6%)	<.0001
ASA	30 (14%)	32 (15.3%)	.16
NSAIDs	57 (25%)	49 (13.9%)	.05

ASA, acetylsalicylic acid; GERD, gastroesophageal reflux disease; NSAIDs, non-steroidal anti-inflammatory drugs; PPI, proton pump inhibitors.
